# Prescribing Variation in General Practices in England Following a Direct Healthcare Professional Communication on Mirabegron

**DOI:** 10.3390/jcm7100320

**Published:** 2018-10-03

**Authors:** Frank Moriarty, Shegufta Razzaque, Ronald McDowell, Tom Fahey

**Affiliations:** 1 HRB Centre for Primary Care Research, Department of General Practice, Royal College of Surgeons in Ireland, 123 St Stephen’s Green, Dublin, D02 YN77, Ireland; sheguftarazzaque@rcsi.ie (S.R.); r.mcdowell@qub.ac.uk (R.M.); tomfahey@rcsi.ie (T.F.); 2 Cancer Epidemiology and Health Services Research Group, Centre for Public Health, Queen’s University Belfast, Belfast BT12 6BA, UK

**Keywords:** pharmacovigilance, drug safety, segmented regression, interrupted time series, variation

## Abstract

Introduction: Pharmacovigilance may detect safety issues after marketing of medications, and this can result in regulatory action such as direct healthcare professional communications (DHPC). DHPC can be effective in changing prescribing behaviour, however the extent to which prescribers vary in their response to DHPC is unknown. This study aims to explore changes in prescribing and prescribing variation among general practitioner (GP) practices following a DHPC on the safety of mirabegron, a medication to treat overactive bladder (OAB). Methods: This is an interrupted time series study of English GP practices from 2014–2017. National Health Service (NHS) Digital provided monthly statistics on aggregate practice-level prescribing and practice characteristics (practice staff and registered patient profiles, Quality and Outcomes Framework indicators, and deprivation of the practice area). The primary outcome was monthly mirabegron prescriptions as a percentage of all OAB drug prescriptions and we assessed the change following a DHPC issued by the European Medicines Agency in September 2015. The DHPC stated mirabegron use was contraindicated with severe uncontrolled hypertension and cautioned with hypertension. Variation between practices in mirabegron prescribing before and after the DHPC was assessed using the systematic component of variation (SCV). Multilevel segmented regression with random effects quantified the change in level and trend of prescribing after the DHPC. Practice characteristics were assessed for their association with a reduction in prescribing following the DHPC. Results: This study included 7408 practices. During September 2015, 88.9% of practices prescribed mirabegron and mirabegron comprised a mean of 8.2% (SD 6.8) of OAB prescriptions. Variation between practices was classified as very high and the median SCV did not change significantly (*p* = 0.11) in the six months after the September 2015 DHPC (12.4) compared to before (11.6). Before the DHPC, the share of mirabegron over all OAB drug prescriptions increased by 0.294 (95% confidence interval (CI), 0.287, 0.301) percentage points per month. There was no significant change in the month immediately after the DHPC (−0.023, 95% CI −0.105 to 0.058), however there was a significant reduction in trend (−0.036, 95% CI −0.049 to −0.023). Higher numbers of registered patients, patients aged ≥65 years, and practice area deprivation were associated with having a significant decrease in level and slope of mirabegron prescribing post-DHPC. Conclusion: Variation in mirabegron prescribing was high over the study period and did not change substantively following the DHPC. There was no immediate prescribing change post-DHPC, although the monthly growth did slow. Knowledge of the degree of variation in and determinants of response to safety communications may allow those that do not change prescribing habits to be provided with additional support.

## 1. Introduction

When medicines are first launched, evidence of drug efficacy and safety may be incomplete, and for approximately 10% of drugs, information about serious risks associated with the drug do not become known until after being released onto the market [1]. The pre-marketing phase based on randomised controlled trials generally involves healthier participants than the general patient population, relatively short durations of follow-up, and sample sizes which only power to detect a difference in the primary efficacy outcome. Post-marketing pharmacovigilance is necessary to monitor benefits and risks based on real-world use. Emerging safety issues identified in post-marketing monitoring may require regulatory action to maintain a favourable risk-benefit ratio. This can involve a change in the terms of a product licence, a direct healthcare professional communication (DHPC) from medicine regulators to healthcare professionals warning of a new adverse effect, caution, or contraindication, or withdrawal of a drug from the market. 

An example of a drug recently subject to a Europe-wide DHPC is mirabegron, licensed for the treatment of overactive bladder (OAB) by the European Medicines Agency (EMA) in December 2012 [2]. It is a beta-3 adrenoreceptor agonist and is the first treatment for OAB with this therapeutic target. Other pharmacological treatment options for OAB such as oxybutynin are antimuscarinic drugs, which carry a risk of anticholinergic adverse effects due to their mechanism of action, such as dry mouth, dizziness, constipation, and cognitive impairment [3]. Mirabegron, as a new active substance, is subject to additional monitoring post-marketing, generally for a period of five years under EMA rules. In July 2015, a review of safety data by the EMA found an increased risk of severe hypertension associated with mirabegron, and cerebrovascular and cardiovascular events such as stroke linked to mirabegron had been reported. The EMA deemed that this required active dissemination regarding the change of use of mirabegron. A DHPC letter was sent to healthcare professionals in September 2015 by European medicine regulators to inform them that mirabegron was contraindicated in patients with severe uncontrolled hypertension (systolic blood pressure ≥180 mmHg or diastolic blood pressure ≥110 mmHg, or both) [4]. The product license was also amended to caution prescribing where systolic or diastolic blood pressure is ≥160 mmHg or diastolic blood pressure ≥100 mmHg respectively.

DHPCs have been shown to be effective in changing prescribing behaviour. The impact of these has been evaluated for a wide range of therapeutics, including selective serotonin reuptake inhibitors, antipsychotics and oral contraceptives [5,6,7], and DHPCs for safety issues with a risk of death and/or disability may have a greater impact on prescribing [8]. However, the extent to which prescribers vary in their response to regulatory safety communications is unknown. An understanding of the degree of variation in and determinants of uptake of DHPCs may allow groups that do not change prescribing to be supported with specific interventions.

This study aims to explore changes in prescribing in general practices in England following a DHPC regarding the safety of mirabegron. 

The objectives were:

To quantify variation between general practitioner (GP) practices in rates of mirabegron prescribing before and after regulatory safety communication

To determine the effect of this safety warning on the level and trend of mirabegron prescribing among general practices in England

To quantify variation between GP practices in response to the regulatory safety communication, and

To identify GP practice factors that explain variations in the response to the regulatory safety communication.

## 2. Methods

### 2.1. Study Design, Setting and Participants

The STrengthening the Reporting of OBservational studies in Epidemiology (STROBE) statement has been used in the reporting of this research [9]. This study utilises an interrupted time series design, the strongest quasi-experimental design to assess the effect of policy or regulatory interventions [10]. 

The setting is English general practice and includes all GP practices in England using prescribing data available from the National Health Service (NHS) Digital platform. This provides monthly statistics of prescribing of different medicines aggregated at the level of GP practices for all practices in England. The study period was January 2014 to March 2017. Atypical practices were excluded, i.e., those with <750 registered patients or <500 patients registered per full time equivalent (FTE) GP, or if there are >5000 registered patients per FTE GP. This is consistent with previous studies utilising administrative GP practice data from the same source [11]. In addition, practices with fewer than 100 prescriptions per month during the 12 months either before or after the DHPC (i.e., from August 2014 to October 2016) were excluded, to ensure that included practices contributed sufficient data in the immediate period before and after the DHPC.

### 2.2. Variables

The primary outcome was prescriptions for mirabegron as a percentage of all prescriptions for drugs to treat OAB.

Characteristics of GP practices which may relate to prescribing include the number of FTE GPs in each practice, the age and sex distribution of GPs, and whether the practice has any registrar GPs (i.e., qualified doctors undertaking specialist training in general practice) [11]. Indicators of quality of care through the Quality and Outcomes Framework (QOF) are available for each practice, including the overall score, as well as indicators relating to specific conditions such as cardiovascular disease. Information is also provided on the practice list size (i.e., the number of patients registered to each practice), and the age and sex distribution of registered patients. Lastly, although no other practice-level patient characteristics are available, the Index of Multiple Deprivation (IMD) of the geographic area a practice is located in was used.

### 2.3. Data Sources/Measurement

Monthly prescribing data relating to mirabegron, OAB drugs and all prescription items were downloaded from the NHS Digital website for the study period. Prescribed products are coded based on their British National Formulary (BNF) classification, and mirabegron (0704020AE) and OAB drug prescribing (070402) were defined using this coding. All drugs listed in BNF section 7.4.2 (Urinary frequency, enuresis, and incontinence) were considered as OAB drugs (see Supplementary Table S1). The number of prescriptions for each product that was dispensed in the specified month is captured in this data. The data relates to NHS prescriptions issued by general practices in England and dispensed in any community pharmacy in the United Kingdom (UK). Prescriptions may be issued by any prescribing staff within practices, including GPs, nurses, and pharmacists and private prescriptions are not included.

Baseline GP practice workforce and registered patient data (i.e., from 2014) for included practices were downloaded from the NHS Digital website and were summarised at the practice level. In addition, QOF indicators were obtained for 2014 and 2015 for overall score and indicators of hypertension and dementia prevalence (which could plausibly explain variation in prescribing of mirabegron and other antimuscarinic OAB drugs, respectively). For deprivation, the IMD for 2015 is provided for geographic areas (lower-level super output areas or LSOA) by the Department for Communities and Local Government. The index captures the following dimensions of deprivation: income, employment, education, health, crime, access to housing and services, and living environment. Practices were assigned the IMD decile of the LSOA they were located in based on their postcode.

As this study used publicly available data aggregated at the GP practice-level, ethical approval was not required.

### 2.4. Analysis

Descriptive statistics are presented for practices which met inclusion criteria. Prescribing patterns were summarised for each month, including the proportion of practices prescribing any mirabegron, mirabegron and OAB prescriptions, and mirabegron as a percentage of OAB prescriptions. We graphed monthly percentiles of mirabegron’s percentage share of OAB prescriptions to describe variation over time. Between-practice variation in prescribing before and after the September 2015 DHPC was assessed using the systematic component of variation (SCV) based on practices between the 5–95th percentiles of mirabegron prescribing. The SCV estimates the true or non-random part of total variation and performs well as a measure of variation [12,13,14]. Variation is classified as either low (less than 3), moderate (between 3 and 5.4), high (between 5.4 and 10), or very high (greater than 10) [12]. In particular, we examined variation in the six months before and after the DHPC and assessed whether the median SCV differed significantly before and after, using the Wilcoxon ranked sum test. Standardised prescribing ratios (mirabegron’s percentage share of all OAB prescriptions in a practice each month divided by the percentage share across all practices each month) were calculated and plotted by month to visually inspect variation. Ratios >1 indicate a higher percentage than average. To examine variation relative to the month of the DHPC, we calculated a rolling average of practices’ mean percentage of mirabegron prescriptions over the previous six months, expressed as a ratio of the practice percentage in September 2015. Deciles and 1st to 9th bottom and top percentiles of these ratios were graphed to assess whether the distribution of practices differed before and after the DHPC. This approach has been used previously to assess variation following guidance being issued regarding tamoxifen use [15].

Interrupted time series studies of policy interventions can be analysed using segmented regression, allowing for the change in level and trend of an outcome following an intervention to be evaluated [10]. A multilevel segmented regression model was fitted to account for repeated monthly observations clustered within practices, with monthly mirabegron percentage as the outcome. Random effects (to allow slope and intercept parameters to vary by practice) were included to determine the change in level and trend of prescribing after the DHPC, using an unstructured covariance matrix. Appropriateness of inclusion of random effects was assessed using the likelihood ratio test for the following parameters: level of prescribing pre-safety warning in August 2015 (intercept), the change in level of prescribing immediately post-warning in October 2015 (change in intercept), the monthly trend in prescribing pre-warning (slope), and the change in the monthly trend post-warning (change in slope). Calendar month (as a fixed effect) and a second order autoregressive function were included to account for seasonality.

Lastly, the estimated practice-specific parameter for each of the random effects was examined to classify practices according to whether their change in level and change in slope parameters represented a significant increase or decrease (i.e., if the estimate’s 95% confidence interval excluded zero). Practice characteristics were examined as predictors of decreases in level or slope using multivariate logistic regression. Characteristics were included as standardised variables (i.e., rescaled to a mean of zero and a standard deviation of one).

## 3. Results

This study included 7408 GP practices, which represents 98.4% of practices in England as of September 2016. At baseline, included practices had a median of 6613 registered patients (interquartile range (IQR) 4072–9919). The mean percentage of patients aged 65 years and over was 16.8% (SD 6.5), and on average their patients were 49.9% female (SD 2.3). Practices had a median four GP FTEs (IQR 2–6.4). On average, 46.5% of GPs in a practice were female (SD 25.9) with 56.2% aged 45 years and over (SD 28.4), while 25.5% of practices had a registrar.

**Figure 1:**
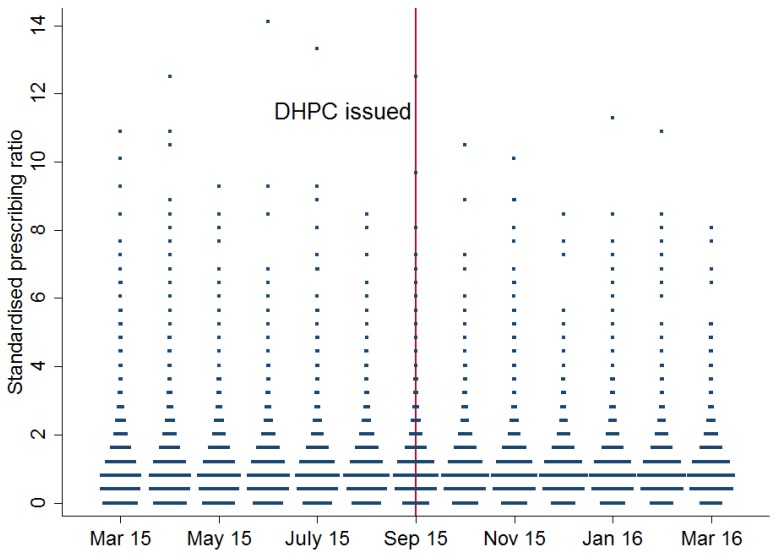
Dot plot of standardised prescribing ratio of mirabegron percentage in the six months before and after the direct healthcare professional communication.

**Figure 2:**
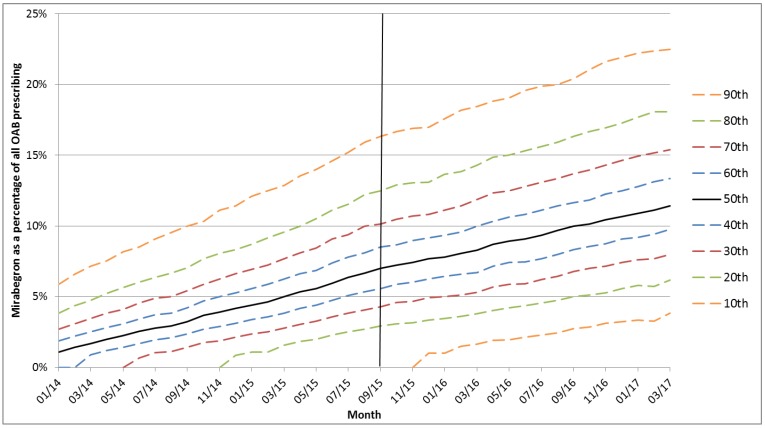
Percentiles of mirabegron prescribing as a percentage of all overactive bladder (OAB) drug prescribing. Line indicates the release of the direct healthcare professional communication. Lower percentiles where mirabegron comprises 0% of OAB prescribing are not included.

**Figure 3:**
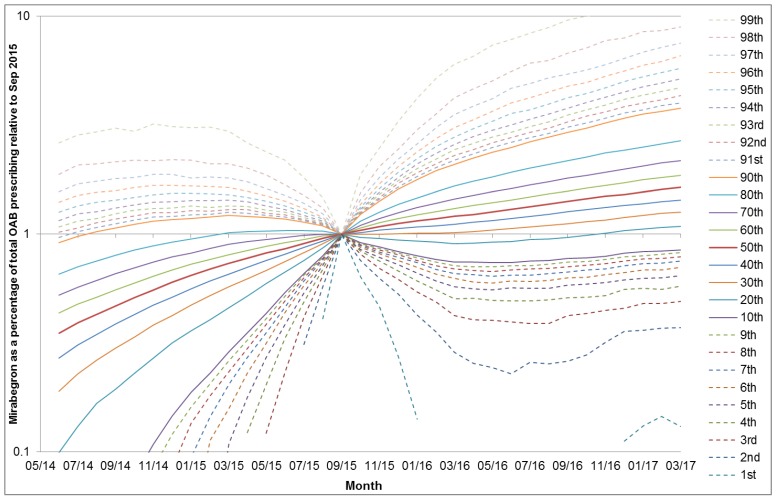
Distribution of practices by mean mirabegron percentage for rolling six-month periods relative to September 2015. Percentiles not shown where rolling mean of mirabegron as a percentage of OAB prescribing is 0%.

During September 2015, 88.9% of practices prescribed mirabegron and mirabegron comprised a mean of 8.2% (SD 6.8) of OAB prescriptions (median 7.0%, IQR 3.6%–11.1%). This corresponded to a mean of 76 OAB prescriptions, of which 6.2 were mirabegron. Variation between practices was classified as very high and the median SCV did not change significantly (*p* = 0.11) in the six months after the September 2015 DHPC (12.68) compared to the six months before (12.04). Among practices with any mirabegron prescribing, standardised prescribing ratios in the six months before and after September 2015 ranged from 0.44–14.1. Figure 1 is a dot plot which illustrates little change in variation over the time period. Figure 2 shows a decile plot of mirabegron percentage, indicating the increasing percentage over time, but little change in the distribution across deciles. Figure 3 shows the distribution of practices by mean mirabegron percentage for rolling six-month periods relative to September 2015, and the distribution was relatively symmetrical with respect to the *Y*-axis before and after the DHPC, suggesting that between-practice variation remained relatively stable.

**Figure 4:**
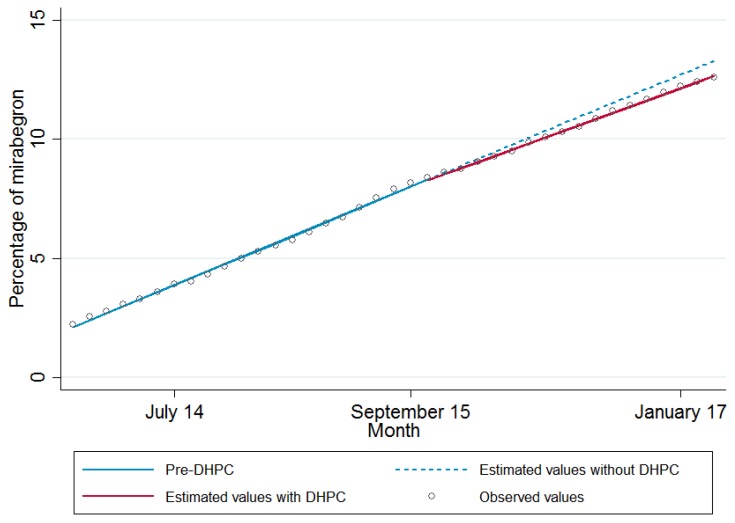
Observed values of mirabegron prescribing and regression estimated values with and without the September 2015 direct healthcare professional communication.

Segmented regression analysis indicates that before the DHPC, there was a trend of 0.294 (95% confidence interval (CI), 0.287, 0.301) percentage points increase per month in the percentage of OAB drugs prescribed as mirabegron (see Table 1 and Figure 4). There was no significant change in percentage of mirabegron prescribing immediately after the DHPC (−0.023, 95% CI −0.105 to 0.058); however, there was a small but significant reduction in trend (−0.036, 95% CI −0.049 to −0.023) after the DHPC. Examining practice-level random effects, 1.8% of practices had an immediate decrease in level of mirabegron prescribing, while 7.1% had a decrease in slope post-DHPC (see Table 2). Increases in level and slope were observed in 1.9% and 4.5% of practices respectively. Estimated mirabegron prescribing for sub-groups of practices with a decrease in level or slope is shown in Figure S1.

Table 3 shows practice characteristics associated with decreases in the level of prescribing and slope. A higher number of registered patients, higher proportion of registered patients aged 65 years and over, and deprivation were all associated with lower odds of an immediate decrease in the level of mirabegron prescribing. Similarly, factors associated with lower odds of a decrease in slope included a higher number of registered patients and deprivation.

## 4. Discussion

Variation in mirabegron prescribing was high and this did not change significantly following a DHPC. At the beginning of the study period, mirabegron was a relatively new medicine to be authorised, having been approved by the EMA in December 2012 and first prescribed on the English market from March 2013. This may be one explanation for the high variation, as practices may adopt prescribing of new products at different rates [16]. At the time of the DHPC, the vast majority of practices were prescribing mirabegron. There was no immediate prescribing change post-DHPC, and although the monthly growth in mirabegron prescriptions did slow, the magnitude of this change was small. Our study could only evaluate aggregate practice-level prescribing and could not separate prevalent and incident use. The decline in the monthly rate of increase in mirabegron could potentially be attributable to reduced incident use, however any change in mirabegron prescribing among at-risk patients may not have been detectable at the practice-level.

Practices with more registered patients and those in more deprived areas were less likely to have a reduction in the level and trend in mirabegron prescribing. This suggests that some practices have a greater capacity to review and amend prescribing if there are fewer patients or less deprivation, or these practices may have fewer or no patients with uncontrolled hypertension and no reason to alter prescribing. Deprivation and inequality are associated with more complex care need through higher prevalence of multimorbidity and polypharmacy [17,18], and poorer health outcomes [19]. In line with the inverse care law, those most in need of care due to inequalities are often least likely to receive it due to reduced capacity of care providers because of the added complexity of care [20,21]. Similarly, the more older patients registered at a practice, the less likely an immediate reduction in the level of mirabegron prescribing. For practices with older patient populations, reluctance to switch to alternative oral OAB drugs (which may have anticholinergic effects) may have contributed to continued growth in mirabegron prescribing. Mirabegron was a second-line therapy in national guidelines at this time and so many patients may already have not responded to or tolerated an alternative OAB drug. No alternatives were recommended in the DHPC, which reflects the limited therapeutic options for OAB available to prescribers caring for older patients, among whom the prevalence of hypertension is high. Alternative medicines for OAB are antimuscarinic, and older adults are particularly susceptible to the anticholinergic effects of such agents (3), and further risks identified in recent years include dementia and cognitive decline [22]. However this evidence is primarily derived from observational research which considers all OAB drugs together, whereas newer agents such as darifenacin may not carry this effect [23]. It is also possible that a doctor and patient for whom mirabegron may be cautioned could decide that the benefits of continuing mirabegron for OAB may outweigh the potential cardiovascular risks. This may be more likely given the relatively small number of cases which prompted the DHPC and that further confirmatory studies have yet to be completed.

Although this appears to be the first study to evaluate variation in response to a DHPC regarding a medication, previous research evaluating indicators of prescribing safety, high-risk prescribing, and antipsychotic prescribing in UK general practice has found variation between practices was similarly high [7,24,25]. Although several studies have evaluated the impact of DHPCs on a range of outcomes, these have not assessed variation between healthcare professions [5,6,26,27]. Evaluating the effectiveness of risk communication has become a focus area in recent years [28], as evidenced by Strengthening Collaboration for Operating Pharmacovigilance in Europe (SCOPE) Joint Action which involves medicine regulators across Europe [29]. An understanding of variation in and the determinants of response to regulatory safety communications among GP practices, and ideally individual general practitioners, may allow for those that do not alter their prescribing to be provided with tailored information and supports to promote safe medication use. There is evidence that there is variation between countries in Europe in GP preferences for the format of safety communications [30]. Despite this, it appears that DHPCs represent the most common source of awareness of medicines’ safety issues among European GPs, pharmacists and cardiologists [31]. Previous research has indicated that such communications have greater impact on non-specialist drugs and for safety issues with a risk of death and/or disability [8] however to date, the relationship between the characteristics of DHPCs and variation in outcomes has not been evaluated. Further research should also evaluate additional interventions to communicate safety information to healthcare professionals in cluster or stepped wedge randomised controlled trials. The only such study to date examined the effect of an additional email on the effectiveness of a DHPC in the Netherlands [32]. Depending on the timing and formats of future DHPCs, this may present opportunities to evaluate the effectiveness of such communications in natural experiments [6].

Systematic reviews illustrate that relatively few evaluations of regulatory actions have been undertaken [5,33,34,35]. Regulatory actions relating to a range of therapeutic agents have been evaluated, with antidepressants being the most commonly examined [33,35]. A substantial proportion of such studies used study designs and analytical approaches which yield low quality evidence of the effects of pharmacovigilance actions i.e., cross-sectional or before and after studies [33,34]. Unlike more methodologically robust interrupted time series studies, these studies do not consider trends in outcome and thus may overestimate the impact of an intervention of interest [10,36]. Therefore, the methodological approach may have an impact on findings, as studies using more robust design tended to report more conservative or mixed impacts of regulatory actions, like the present study [5,33,34].

Evaluating medication utilisation using prescribing or dispensing data is just one way of evaluating such regulatory actions. Other quantitative evaluation could measure changes in adverse outcomes relating to uncontrolled hypertension or cerebrovascular events in the case of mirabegron, or unintended consequences such as inappropriate switching to another OAB drug. Recent proposals have outlined a framework approach to evaluation, including quantitative and qualitative analysis of tradition and social media uptake of the communications, qualitative research with healthcare professionals and patients, as well as more traditional quantitative measures of process and outcomes [37]. Behaviour change and implementation science is a growing area of focus for regulatory bodies in pharmacovigilance and risk minimisation programs [38]. This reflects that moving from awareness of a regulatory safety communication to implementation in clinical practice is complex, with decay at each step in the process [39]. Similarly, the use of complex interventions to support adoption of regulatory safety warnings may increase their impact. For example, this could involve integrating emerging safety communication within computer decision support systems in electronic health records to flag warnings relevant to specific patients during clinical workflow. However, evidence on the effectiveness of computer warnings is mixed, and requiring a reason to override messages may improve effectiveness at the expense of potential alert fatigue [40]. Frameworks such as the adoption of innovation (i.e., innovation, communication channels, time and adoption process, and social systems) could be considered by regulatory agencies to optimise scale-up, spread, and uptake of regulatory actions and communications [41].

This study has a number of strengths. It appears to be the first to assess the impact of the DHPC on mirabegron’s cardiovascular risks on utilisation patterns of OAB drugs in a large primary care cohort. We have also used the most robust method possible in evaluating temporal changes in prescribing. A limitation of this study is the lack of patient-level data, which prevented analysis of mirabegron prescribing among those patient groups affected referred to in the DHPC. It is possible that prescribers may have reduced use of mirabegron in at-risk populations, which may not be detectable with a concurrent rise in mirabegron prescribing to other patients in the practice. Lack of patient-level data also precluded analysis of patient-level characteristics and their association with cessation of mirabegron among prevalent at-risk patients. All of the characteristics examined were at the practice level, with the exception of deprivation of the practice area. While this may indicate the deprivation of the practice populations, there is potential for ecological bias in that registered patients may not have been deprived despite the practice being located in a deprived area. We were also unable to examine patient-level changes in prescribing to determine whether reductions in mirabegron use were appropriately targeted at patients most at risk of cardiovascular harms. Inappropriate switching to other OAB drugs in patients who already had a high anticholinergic burden as an unintended consequence could have resulted in increased net harm. Despite these limitations, this appears to be the first study to evaluate variation between GP practices in response to a DHPC, which may be an important consideration for future pharmacovigilance research.

## 5. Conclusions

While variation in healthcare has received much attention in recent decades, this has not extended to variation in response to regulatory safety communications regarding medications. As medicine regulators develop further strategies to improve the impact of DHPCs on clinical practice, heterogeneity between prescribers in response to such warnings will become an important consideration.

## Figures and Tables

**Table 1:** Segmented regression analysis of mirabegron prescribing as a percentage of all overactive bladder drug prescribing.

Parameters	Adjusted Coefficient * (95% Confidence Interval)	*p* Value
Monthly trend (slope)	0.294 (0.287 to 0.301)	<0.001
Change in monthly trend post DHPC	−0.036 (−0.049 to −0.023)	<0.001
Level of prescribing at point of DHPC (intercept)	8.30 (8.16 to 8.44)	<0.001
Change in level immediately post DHPC	−0.023 (−0.105 to 0.058)	0.574

* Adjusted for calendar month. DHPC = direct healthcare professional communication.

**Table 2:** Number and percentage of practices by level and slope of mirabegron prescribing post direct healthcare professional communication.

	*n* (%)	
	Change in Level	Change in Slope
Decrease ^a^	133 (1.8)	529 (7.1)
No change	7133 (96.3)	6545 (88.4)
Increase ^b^	142 (1.9)	334 (4.5)

^a^ Decrease defined as a practice-level random effect for level/slope where the upper bound of the 95% confidence interval is less than zero. ^b^ Increase defined as a practice-level random effect for level/slope where the lower bound of the 95% confidence interval is greater than zero.

**Table 3:** Practices characteristics associated with a decrease in level of mirabegron prescribing or slope post DHPC.

	Outcome of a Decrease in Level ^a^		Outcome of a Decrease in Slope ^a^	
	Adjusted Odds Ratio (95% Confidence Interval)	*p* Value	Adjusted Odds Ratio (95% Confidence Interval)	*p* Value
GP workforce characteristics				
GP full time equivalents	0.93 (0.52 to 1.66)	0.811	0.83 (0.63 to 1.09)	0.177
Proportion of female GPs	1.10 (0.95 to 1.28)	0.189	1.05 (0.97 to 1.15)	0.238
Proportion of GPs aged 45 and over	1.10 (0.94 to 1.30)	0.236	1.04 (0.95 to 1.14)	0.344
Any registrars ^b^	0.83 (0.60 to 1.16)	0.283	1.08 (0.95 to 1.23)	0.233
Patient characteristics				
Number of registered patients	0.40 (0.24 to 0.67)	<0.001	0.57 (0.45 to 0.73)	<0.001
Proportion of female patients	0.90 (0.80 to 1.02)	0.091	0.97 (0.89 to 1.05)	0.464
Proportion of registered patients aged 65 and over	0.52 (0.36 to 0.75)	0.001	0.88 (0.74 to 1.06)	0.180
Other practice characteristics				
Overall QOF score percentage ^c^	0.91 (0.80 to 1.05)	0.188	0.97 (0.90 to 1.06)	0.525
Hypertension prevalence	1.07 (0.81 to 1.42)	0.619	0.90 (0.77 to 1.04)	0.154
Dementia prevalence	0.83 (0.53 to 1.31)	0.428	0.83 (0.67 to 1.04)	0.102
Index of multiple deprivation (deciles)	1.30 (1.07 to 1.59)	0.010	1.22 (1.10 to 1.35)	<0.001

^a^ Decrease defined as a practice-level random effect for level/slope where the upper bound of the 95% confidence interval is less than zero. ^b^ A registrar is a doctor who has completed foundation training and is training in the specialty of general practice. ^c^ Quality and Outcomes Framework (QOF) score reflects a practice’s performance against a range of indicators to describe best practice.

## Data Availability

The source data used in this study is publicly available at https://digital.nhs.uk/. The dataset and the analytical code relating to this study are available from: http://doi.org/10.5281/zenodo.1409023

## Supplementary Materials

The following are available online at http://www.mdpi.com/2077-0383/7/10/320/s1, Figure S1: Estimated values for proportion of mirabegron prescribing without and with the direct healthcare professional communication (DHPC), graphed by significant reduction in slope or level post-DHPC., Table S1: All agents considered as overactive bladder drugs.
