# Supplementary material for: Prescribing Variation in General Practices in England Following a Direct Healthcare Professional Communication on Mirabegron
Source: J Clin Med. 2018 Oct 3;7(10):320. doi: 10.3390/jcm7100320 (PMC6210595; doi:10.3390/jcm7100320)
Supplement: Supplementary file 1 [file jcm-07-00320-s001.pdf]

Supplementary materials

Table S1. All agents considered as overactive bladder drugs.

**Overactive bladder drugs**
Darifenacin
Duloxetine
Flavoxate hydrochloride
Fesoterodine fumarate
Mirabegron
Oxybutynin hydrochloride
Propantheline bromide
Solifenacin succinate
Tolterodine tartrate
Trospium chloride

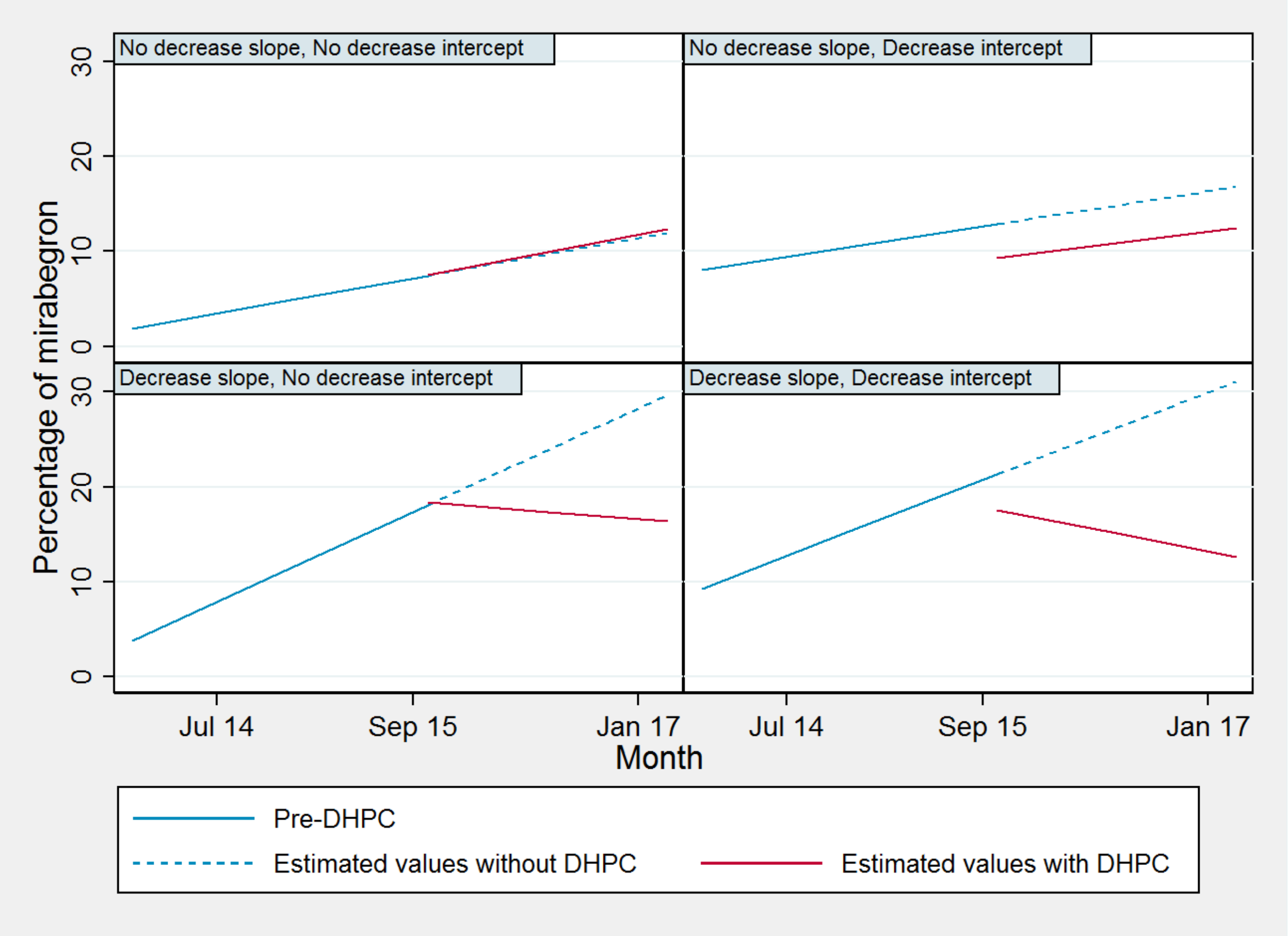

Figure S1. Estimated values for proportion of mirabegron prescribing with and without the direct healthcare professional communication (DHPC), graphed by significant reduction in slope or level post-DHPC.
